# A Single-Operator Push-Cart Multi-Beam LiDAR Platform for Multi-Trait Field Phenotyping

**DOI:** 10.3390/s26144444

**Published:** 2026-07-13

**Authors:** Matthew H. Siebers, Caleb M. T. Sindic, Michael Boettcher

**Affiliations:** USDA-ARS Dairy Forage Research Unit, Madison, WI 53706, USA

**Keywords:** LiDAR, biomass, phenomics

## Abstract

Here, we present a single-operator push-cart platform equipped with a 16-beam LiDAR. A push-button interface controls data acquisition, and the data processing pipeline removes ground points, filters noise, performs 5-cm voxelization, and produces plot-level canopy metrics. We validated biomass estimation in hairy vetch (*Vicia villosa*) and corn (*Zea mays*) leaf- and whole-plant thinning experiments. In vetch, voxelized estimation of plant volume correlated strongly with destructively measured biomass (r^2^ = 0.88), showing that the multi-beam LiDAR can produce biomass estimates comparable to previously reported methods. In corn, comparisons of perpendicular (0°) and multi-angle LiDAR beams showed significantly greater voxel counts in the upper canopy when angled beams were used (beam angle × height interaction, *p* < 0.001), demonstrating that multi-beam scanning provides greater penetration into the upper canopy than a single perpendicular scan plane. We also extended the suite of LiDAR-derived traits to include apparent leaf area index (LAI), mean tilt angle (MTA), persistent homology-based stand density, and plot-bounded foliage area density (FAD). The persistent homology algorithm distinguished between leaf-removal and plant-removal treatments (removal type × removal amount, *p* = 0.0039). LiDAR-derived LAI has been used to estimate canopy leaf area, but gap-fraction approaches do not fully exploit the ability of LiDAR to resolve distance. Plot-bounded FAD used ray length and interception distance within defined plot volumes and was more sensitive to plot-level treatments than apparent LAI or MTA, detecting differences associated with both the removal amount and removal type. These results show that a robust, portable, multi-beam LiDAR cart can reproduce plot-level canopy measurements and improve trait especially in research-sized plots.

## 1. Introduction

Plant breeding progress is increasingly constrained not by genotyping capacity, but by the ability to measure complex structural traits in the field. Biomass, canopy architecture, and vertical leaf area distribution remain central determinants of yield and stress tolerance, yet researchers still commonly assess them using destructive harvests or visual scoring [1,2,3]. High-throughput phenotyping has expanded rapidly in controlled environments, but scalable, plot-level field tools that are accurate, rapid, and biologically interpretable remain limited [4,5]. For crops such as maize and forage legumes, where canopy density and vertical stratification drive light interception and productivity, improved structural sensing is particularly important.

LiDAR provides a direct measurement of three-dimensional canopy structure and has emerged as one of the most promising tools for non-destructive biomass estimation. In wheat, maize, cotton, and other row crops, proximal LiDAR has been used to estimate plant height, leaf area distribution, and above-ground biomass, often with strong correlations to destructive measurements [6,7,8,9,10,11,12]. In maize specifically, terrestrial laser scanning has enabled detailed reconstruction of stalk and leaf architecture and extraction of phenotypic traits relevant to canopy function [10,12,13,14,15,16]. Structural information derived from LiDAR point clouds is commonly summarized using voxelization, which discretizes space into volumetric units and provides robust estimates of canopy occupancy [6,8,11,17,18,19]. Voxel-based metrics have shown strong predictive relationships with biomass across crop types and growth stages. Additionally, voxel distributions can also be resolved vertically, enabling analysis of canopy stratification rather than total volume alone.

Despite these advantages, most agricultural LiDAR systems used in field phenotyping rely on single-beam rotating planar scanners. These instruments sweep a two-dimensional profile perpendicular to the direction of travel and reconstruct 3D structure through forward motion [7,12,17]. Although effective, this architecture limits vertical sampling to a single instantaneous beam angle. In dense canopies such as maize or vetch, lower foliage can occlude upper canopy elements and reduce structural representation at height. The limitation is therefore not sensor range, but sampling geometry.

Multi-beam time-of-flight sensors address this constraint by using multiple distinct laser channels that fire simultaneously across a fixed vertical field of view. Each channel operates as an independent ranging unit and provides concurrent measurements at multiple angles during forward motion. This architecture, commonly used for mobile mapping and navigation, enables simultaneous vertical sampling without requiring multiple passes or mechanical tilting [20]. Similar mobile 3D sensing and sensor-fusion approaches have also been developed outside agriculture, including robotic structural inspection systems that combine LiDAR, camera calibration, and 3D reconstruction for automated crack detection [21]. Occlusion and beam geometry are known to influence LiDAR-derived structural metrics, and studies examining voxel size, angular distribution, and sampling bias have shown that canopy reconstructions are sensitive to beam configuration and viewing geometry [19,20]. In crops with layered foliage, multiple angular scans may provide additional penetration pathways to the upper canopy and reduce occlusion.

LiDAR point clouds can also support additional canopy traits beyond voxelized volume. Stand count can be estimated from the spatial organization of lower-canopy points using topology-based approaches such as persistent homology. Gap-fraction methods developed for hemispherical canopy analyzers can be adapted to LiDAR data to estimate apparent leaf area index (LAI) and mean tilt angle (MTA) [22,23,24,25]. However, conventional gap-fraction approaches do not fully use one of the central advantages of LiDAR: each return has a known distance. Distance information allows each ray to be evaluated within a defined plot volume and makes it possible to estimate plot-bounded foliage area density (FAD) from ray path-length and interception location. Together, these traits extend LiDAR analysis from canopy volume alone toward a broader evaluation of stand density, canopy closure, canopy leaf angle, and vertical foliage distribution.

We evaluated the platform in two biological systems: (i) a hairy vetch nursery was scanned to test the relationship between voxelized canopy volume and destructively measured biomass, and (ii) a maize thinning experiment used to examine whether angled LiDAR beams improves detection of upper canopy structure relative to the perpendicular beam alone. In the maize experiment, we also tested whether the same LiDAR scans could support additional structural traits, including persistent homology-based stand count, apparent LAI, MTA, and plot-bounded FAD. We hypothesized that voxelized canopy volume would strongly predict biomass in vetch and that multi-beam sampling would yield greater voxel occupancy in upper canopy deciles in maize because of reduced occlusion bias. We also expected that plot-bounded FAD would be more sensitive to leaf- and whole-plant-removal treatments than gap-fraction traits because FAD constrains the analysis to the experimental plot volume. By combining multi-beam scanning, plot-based data collection, and multiple LiDAR-derived canopy traits, this study establishes a practical tool for high-throughput phenotyping in row crops and forage systems. Further testing is required to establish the limits of its applications.

## 2. Materials and Methods

### 2.1. LiDAR Cart Platform

**Figure 1:**
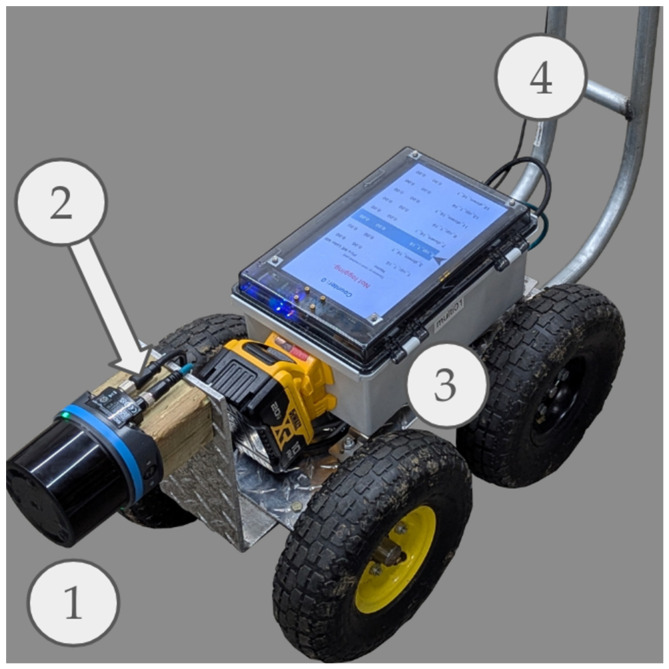
Single-operator push-cart platform for plot-scale LiDAR phenotyping. The cart carried a (1) SICK multiScan136 multi-beam time-of-flight LiDAR mounted horizontally 0.39 m above the ground, (2) an inertial measurement unit for roll and pitch correction, and a wheel encoder for distance tracking. A Raspberry Pi 5 (3) recorded the LiDAR data stream, while a Raspberry Pi Pico 2 recorded encoder counts and IMU orientation. Both data streams were synchronized using a shared pulse-per-second signal from a DS3231 real-time clock. Data collection was controlled with a handle-mounted (4) logging button and a custom Python v3.11 interface that selected the experiment, started and stopped scans, and saved paired LiDAR and microcontroller files for each plot.

A single-operator push-cart platform was developed for proximal-, plot-, and plant-scale LiDAR phenotyping (Figure 1). The aluminum cart frame was 70 cm long from the front of the LiDAR mount to the handle mount, 33 cm wide from the outside of one wheel to the other, and 42 cm tall from the ground to the center of the LiDAR. The cart used 10-inch pneumatic wheels mounted on 3D-printed axle supports. The handle was pinned in place during operation and removed for transport. A rechargeable, 20 V lithium-ion battery powered all electronics. A single battery charge powered the system for approximately 6–8 h of field operation.

The cart carried three primary sensors: a multi-beam LiDAR, an inertial measurement unit, and a wheel encoder. The LiDAR sensor was a SICK multiScan136 (SICK AG, Waldkirch, Germany) time-of-flight sensor mounted horizontally, 0.39 m above the ground. The sensor was mounted on its side and contained 16 scanning planes. Relative to the direction of cart travel, 10 scan planes were oriented forward and sampled from 2.5° to 42.5° ahead of the cart. One scan plane was oriented perpendicular to the direction of travel and is referred to here as the zero-degree scan plane. The remaining five scan planes sampled from 2.5° to 22.5° behind the cart. Each scan plane rotated at 20 Hz. The sensor had an operating range of 0.05–60 m and reported both distance and return intensity for each detected object.

An incremental rotary encoder (E6B2-CWZ6C, OMRON Corporation, Kyoto, Japan) coupled to one wheel of the front axle measured distance. The encoder produced 1024 pulses per revolution, and monitoring both rising and falling edges of the A and B channels yielded 4096 counts per wheel rotation. Encoder calibration used 20 repeated 20-ft pushes of the cart. The calibration runs averaged 30,961 ± 49 counts per 20 ft, corresponding to 5080 counts m^−1^ and 0.197 mm count^−1^. This produced an estimated error of approximately 1.6 mm m^−1^.

A BNO055 inertial measurement (Adafruit Industries, New York, NY, USA) unit mounted inside the electronics enclosure near the front of the cart measured cart orientation. The IMU logged Euler angles for roll, pitch, and yaw at 100 Hz. Roll and pitch corrected scanning geometry during post-processing for changes in cart position caused by uneven terrain.

### 2.2. Data Acquisition and Synchronization Hardware

A Raspberry Pi 5 (Raspberry Pi Ltd., Cambridge, UK) and a Raspberry Pi Pico 2 microcontroller (Raspberry Pi Ltd., Cambridge, UK) mounted inside the electronics enclosure controlled data collection. The Raspberry Pi 5 recorded the LiDAR data stream, and the Pico 2 recorded encoder counts and IMU orientation. Both devices connected to a DS3231 real-time clock module (Adafruit Industries, New York, NY, USA) that emitted a 1 Hz square-wave pulse. Both devices recorded this shared pulse-per-second signal, which aligned the LiDAR and Pico data streams during post-processing. In a 50-s test, the clock module produced pulses at 1.000000 ± 0.000030 s intervals, providing a stable synchronization reference for combining the two independently logged data streams.

A custom Tkinter-based Python (v3.11) interface controlled field data collection. The interface included an experiment-selection menu, a logging menu, and a backup and shutdown menu. Before scanning, the user selected an experiment file containing the plot list and scan order. The software then created a dated folder for the selected experiment. During logging, the interface displayed the plots to be scanned and the size of the data files being generated. Pressing the logging button on the cart handle started data collection for the selected plot, and pressing the button again stopped logging and saved the LiDAR and microcontroller files.

At the start of each scan, scan-relative timing and position counters were reset. The pulse-per-second count and encoder increment were zeroed, allowing each LiDAR and Pico file to be aligned relative to the beginning of that scan. Zeroing these counters at scan start, rather than calculating differences after scan completion, reduced the number of characters written to the data files and simplified post-processing. The LiDAR file contained the LiDAR timestamp, beam angle, distance, return intensity, and pulse-per-second count. The Pico file contained the microcontroller timestamp, encoder count, roll, pitch, yaw, and pulse-per-second count.

### 2.3. Point-Cloud Generation and Plot Splitting

All post-processing used custom Python (v3.11) scripts that could run from a central computer. LiDAR and Pico data were synchronized using the shared pulse-per-second counter recorded in both data streams. Timestamps were then converted to a common pulse-per-second centered time scale, and encoder position and IMU orientation were linearly interpolated onto each LiDAR return using samples from the corresponding and adjacent pulse-per-second windows. This assigned an encoder-derived cart position and roll/pitch orientation correction to each LiDAR point.

LiDAR returns were converted from polar coordinates into Cartesian coordinates and expressed in a cart-centered coordinate system, where X represented lateral distance from the cart, Y represented height above the ground, and Z represented distance along the direction of travel. Encoder counts were converted to actual distance using the wheel calibration. Roll and pitch corrections compensated for cart motion across uneven ground. The LiDAR mounting height was added to place the point cloud in ground-referenced coordinates.

After point-cloud generation, scans were split into the appropriate dimensions using the plot list and user-defined configuration settings. Plot filenames indicated whether each file represented a single plot, a continuous multi-plot scan, or a scan with experimental rows on both sides of the LiDAR. The configuration file specified the plot width and the start and end positions retained for analysis. For continuous scans, the encoder position was used to divide the scan into plot-length sections. When adjacent rows were scanned simultaneously, points were assigned to left or right plots based on their lateral position relative to the LiDAR centerline. Plot names also encoded scan direction and row identity, allowing the parser to assign points to the correct plot even when the cart was pushed in opposite directions on different passes. This step produced plot-level point clouds containing X, Y, and Z coordinates and LiDAR return intensity. Additional ray metadata, including scan angle, beam angle, range, timestamp, encoder position, and interpolated IMU orientation, were retained for downstream trait calculations.

### 2.4. Point-Cloud Filtering and Trait Extraction

Plot-level point clouds were filtered and analyzed using settings defined in an experiment configuration file. The configuration specified plot geometry, including plot boundaries, start and end positions, minimum range, and optional height limits. It also allowed individual traits to be toggled on or off and their parameters to be adjusted. Configurable traits included canopy height (m), leaf area index (LAI; dimensionless), mean tilt angle (MTA; degrees), apparent foliage area density (FAD; m^2^ m^−3^), topology-based stand count, and voxel count.

When enabled, LiDAR return intensity was normalized by scan-angle to reduce beam-specific intensity differences. Normalization calculated a within-angle z-score for return intensity and applied an exponential transform so that returns brighter than the angle-specific mean received greater weight while low-intensity returns were compressed toward zero.

Trait-level operations were applied sequentially in the order listed in the configuration file. These operations could include height-range filtering, bilateral smoothing of normalized return intensity, scalar-range filtering, statistical outlier removal, topology-based stand-count estimation, and voxel counting. Because each enabled operation either modified the current plot-level point cloud or calculated a trait from it, the order of operations determined which filtered point cloud was used for each downstream calculation. For example, stand-count estimation used a point cloud filtered to the lower canopy and by normalized return intensity, so that persistent homology was applied primarily to stem-like structures rather than the full canopy.

Voxel-based canopy volume was calculated, when enabled, by discretizing the filtered point cloud into 0.05-m cubic voxels and counting the occupied voxels within each plot. Voxel count served as a plot-level structural metric and as the primary LiDAR predictor of vetch biomass. Other enabled traits were calculated from the same plot-level processing framework, including LAI, mean tilt angle, apparent foliage area density, canopy height, and topology-based stand count.

### 2.5. LiDAR Gap Fraction and LAI

LiDAR-derived LAI was calculated from angular gap fractions using a LiDAR-adapted gap-fraction approach introduced previously for high-throughput phenotyping method comparison [22]. The approach is analogous to optical canopy analyzers such as the LAI-2000 and LAI-2200C, which estimate canopy structure from the angular dependence of gap fraction [23,24,25]. Candidate LiDAR rays were grouped by zenith angle, and gap fraction was estimated as the proportion of rays that were not intercepted by vegetation. LAI was then estimated from the angular dependence of gap fraction following the general theory used by indirect optical LAI methods, in which directional probabilities of non-interception are related to canopy amount and projection geometry [23,24,25].

### 2.6. Mean Tilt Angle Estimation

Mean tilt angle was estimated from the angular dependence of LiDAR gap fraction using the same framework as LAI [22,23,24,25]. Gap fractions were calculated across zenith-angle classes, and the observed pattern was compared with the expected angular dependence of canopy interception under a leaf-angle projection model. The mean tilt angle parameter was estimated as the value that minimized the difference between observed and expected gap-fraction behavior across angle classes. This produced a plot-level estimate of apparent, average canopy-element inclination. As with LiDAR-derived LAI, this trait was interpreted as an apparent structural metric because LiDAR beams interact with stems, leaves, and other canopy elements rather than leaves alone.

### 2.7. Plot-Constrained Foliage Area Density

Foliage area density (FAD) was calculated using a plot-bounded ray-tracing approach that retained information about the vertical distribution of canopy LiDAR interception. The goal was to estimate the amount of canopy material within defined vertical layers while accounting for the path length of each LiDAR ray through the plot volume.

For each plot, a three-dimensional bounding box was defined by the plot dimensions from the experimental configuration file. The X bounds represented the lateral plot width, the Z bounds represented the plot length, and the Y bounds represented the vertical canopy space above the ground. The upper Y bound was determined by the canopy height. Canopy height was estimated for each plot from the 99th percentile of the Grubbs-filtered *Y*-axis point distribution. The vertical canopy range was subdivided into horizontal layers using an arbitrary but fixed layer thickness of 0.25 m.

For each LiDAR ray, ray–box intersection was used to compute entry and exit points through each layer’s plot-bounded volume. Only the portion of a ray that physically passed through a given layer within the plot boundaries contributed to that layer’s estimate. Rays that did not intersect the plot volume were excluded, preventing returns and path lengths originating outside the plot from contributing to the estimate.

Within each layer, foliage area density was estimated as a path-length-weighted contact frequency:

FAD*_i_* = N*_i_*/∑*_j_*(G*_j_* · l*_ij_*)

where N_i_ is the number of interceptions occurring inside layer *i* within the plot boundaries, *l_ij_* is the path length of ray *j* through layer *i*, and *G_j_* is the mean projection coefficient of canopy elements onto the plane normal to ray *j*. Under an assumption of randomly oriented, spherical canopy elements, *G_j_* was set to 0.5. A ray intercepted in a shallower layer did not contribute path length or interceptions to deeper layers; this restricted each layer’s estimate to rays that actually sampled that portion of the plot volume. Layers containing fewer than a minimum number of valid rays were excluded from the profile.

Integrated FAD was calculated by summing layer-level FAD multiplied by layer thickness across the canopy profile:

Integrated FAD = ∑_i_(FAD*_i_* · Δy*_i_*)

where FAD*_i_* is the apparent foliage area density of layer *i*, *Δ*y*_i_* is the thickness of layer *i*, and *∑_i_* indicates summation across all vertical layers included in the analysis. For this paper, integration was performed over a fixed vertical window, 0.10–1.85 m, common to all plots so that integrated FAD was comparable across plots and was not confounded by plot-to-plot differences in maximum canopy height. Integrated FAD is a leaf-area-index-like quantity, expressed as area per unit ground area, m^2^ m^−2^. However, unlike whole-canopy LAI, integrated FAD retains the vertical FAD profile and is constrained to the plot volume.

### 2.8. Persistent Homology Stand-Count Estimation

Plant count in the corn-thinning experiment was estimated using a persistent homology-based algorithm (Siebers et al., in review). Before topology analysis, point clouds were filtered by height and return intensity. Points between 0.1 and 1.0 m above the ground were retained. LiDAR return intensity was normalized by scan angle, and the scalar intensity threshold was selected by visual assessment to maximize the difference between high- and low-intensity points.

After height and intensity filtering, points were projected onto a horizontal X-Z grid. The algorithm identified connected components that persisted across a fixed persistence threshold. In this study, a point-cloud object was classified as a corn stalk when its persistence exceeded 0.35 of the point-cloud height. Retained components were interpreted as plant locations, and their centroids were saved as detected plant positions. Plot-level stand count was calculated as the number of detected plants divided by plot length and reported as plants m^−1^. The same preprocessing and persistence settings were applied to all plots so that treatment comparisons reflected differences in canopy structure rather than changes in algorithm tuning.

### 2.9. Experimental Data

The LiDAR cart was evaluated in two field experiments at the USDA-ARS Dairy Forage Research Center near Prairie du Sac, WI, USA.

The first experiment was a hairy vetch (*Vicia villosa*) nursery used to evaluate LiDAR-derived canopy volume as a predictor of biomass. Hairy vetch plants were grown in rows spaced 1.52 m apart, with individual plants spaced 1.52 m apart within rows. LiDAR scans and destructive harvests were performed on 23 May 2025. Individual plants were harvested after scanning, and dry matter was used as the reference biomass measurement. Plot-level point clouds were split to the known plant spacing, filtered, voxelized, and compared with destructively measured biomass.

The second experiment was a corn-thinning experiment used to test whether LiDAR-derived traits could distinguish plant removal from leaf removal. Corn (*Zea mays*) was planted on 7 June 2023. Plots were 3.05 m long and contained four rows spaced 0.76 m apart, with plants spaced approximately 0.15 m apart within rows. The experiment included a control and six removal treatments. Plant-removal treatments removed 25%, 50%, or 75% of plants from the plot by cutting entire plants at the base and removing them from the plot. Leaf-removal treatments removed 25%, 50%, or 75% of leaves from each plant, beginning at the base. For 25% leaf removal, every fourth leaf was removed; for 50% leaf removal, every other leaf was removed; and for 75% leaf removal, three of every four leaves were removed. Treatments were assigned in a completely randomized design. Each removal treatment had three replicate plots, and the control had six replicate plots.

### 2.10. Multi-Beam and Single-Beam Canopy Sampling Comparison

To evaluate whether multi-beam LiDAR improved canopy sampling relative to a conventional, perpendicular single-scanning plane, point clouds from the corn-thinning experiment were separated into two beam-angle classes. The zero-degree scan plane, oriented perpendicular to the direction of cart travel, served as the single-beam comparison because it approximated the geometry of a conventional two-dimensional line-scanning LiDAR system. The remaining non-zero, angled scan planes were grouped as the multi-angle class.

Because the multi-angle class contained more scan planes and therefore more points than the zero-degree class, the multi-angle point cloud for each plot and scan was randomly downsampled to match the point density of the corresponding zero-degree point cloud. Both point clouds were then voxelized using the same 0.05-m voxel size. Voxel counts were summarized by canopy height percentile, and the resulting height profiles tested whether angled beams increased voxel number in different portions of the canopy.

### 2.11. Statistical Analysis

For the hairy vetch experiment, the relationship between LiDAR voxel count and destructively measured dry biomass was evaluated using a Pearson’s correlation coefficient.

For the corn multi-beam analysis, voxel counts were analyzed using an analysis of variance with treatment, beam-angle, canopy height, and their interactions as fixed effects. Beam-angle tested the voxel density between the zero-degree scan plane and the downsampled angled scan planes. Canopy height tested the effect of vertical position within the canopy. The beam-angle × height interaction tested whether multi-angle LiDAR sampling changed the vertical distribution of detected canopy volume relative to the zero-degree scan plane.

For the corn trait analysis, plot means were calculated from five repeated scans before statistical testing. Separate analyses were performed for topology-derived stand count, LiDAR LAI, mean tilt angle, and integrated FAD. Treatment effects were evaluated by analysis of variance, and Tukey’s honestly significant difference test was used for multiple comparisons among treatment means. Differences were considered significant at α = 0.05.

Repeatability of LiDAR-derived traits was evaluated using the five repeated scans collected for each plot, following the broader principle that phenotyping method comparisons should report measurement variability in addition to treatment means [22].

## 3. Results

### 3.1. Synchronized Scans Correlated with Whole-Plant Volume

The LiDAR cart successfully collected synchronized LiDAR, encoder, and IMU data in both the hairy vetch nursery and the corn-thinning experiment. In the vetch nursery, approximately 1300 plants were scanned in 45 min, producing 22.5 GB of combined LiDAR and Pico data across all plots. Raw LiDAR and positional data were synchronized using the shared pulse-per-second signal, converted into plot-level point clouds, filtered to remove ground returns below 0.1 m, and voxelized using 0.05 m cubic voxels.

**Figure 2:**
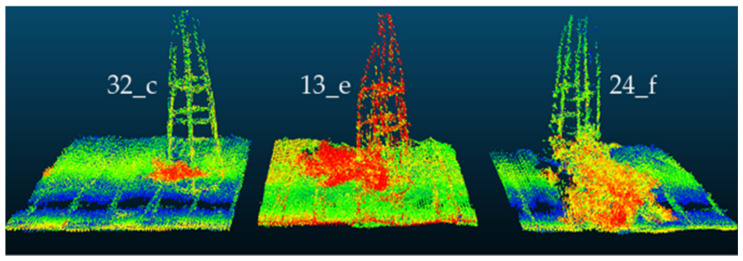
**Representative 3D LiDAR reconstructions of hairy vetch plots scanned on 23 May 2025.** Plots 32_c, 13_e, and 24_f were selected to represent low, intermediate, and high biomass levels based on destructive harvest measurements. Each reconstructed plot was clipped to a 5 ft × 5 ft area. Point color represents relative LiDAR return intensity. Plant points generally had higher relative reflectivity than the black tarp used as the ground surface in the nursery.

**Figure 3:**
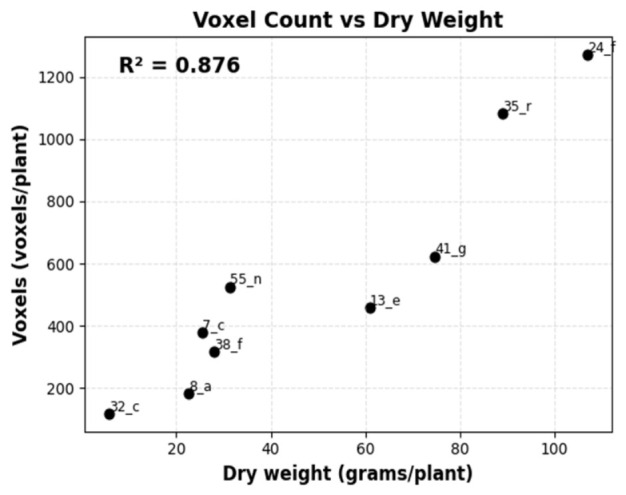
Relationship between LiDAR-derived voxel count and destructively measured dry biomass in hairy vetch. Individual plants were scanned in the field on 23 May 2025, and destructively harvested the same day. LiDAR point clouds were synchronized with encoder and IMU data, split into plot-level scans, filtered to remove ground returns below 0.05 m, and voxelized using 0.05-m cubic voxels. Each point represents one harvested plant. The regression line shows the relationship between occupied voxel count and dry matter biomass. LiDAR voxel count explained 87.6% of the variation in destructively measured dry biomass (r^2^ = 0.876), indicating that voxelized canopy volume captured biologically meaningful differences in plant size.

Representative vetch point clouds showed clear differences in plant size across the biomass range. Of the plants that were destructively harvested, plots 32_c, 13_e, and 24_f (Figure 2) represented low, intermediate, and high values of biomass. Each plot was split to 5 ft in length and 5 ft in width. Return intensity helped separate plant material from the black ground-cover tarp, as plant points generally had lower relative reflectivity than the tarp surface. Voxel count was associated with destructively measured vetch dry biomass (r^2^ = 0.876; Figure 3).

**Figure 4:**
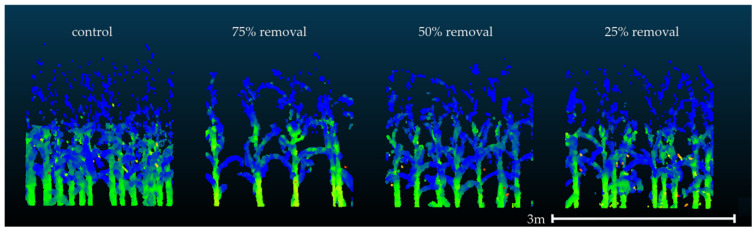
Representative 3D reconstructions of LiDAR point clouds from the corn-thinning experiment. Plots were 10 ft long and consisted of four 30-inch-spaced rows. Treatments included either plant removal or leaf removal. Control plots had no plants or leaves removed. In plant-removal treatments, entire plants were cut at the base and removed from the plot. In leaf-removal treatments, leaves were removed for each plant, beginning at the base: every fourth leaf for 25% removal, every other leaf for 50% removal, and three of every four leaves for 75% removal. The reconstructions shown here represent the control, 25% plant removal, 50% plant removal, and 75% plant-removal treatments. Colors represent the square-root transformation of normalized LiDAR remissivity.

Raw LiDAR and positional data were also successfully combined in the corn-thinning experiment, producing coherent 3D reconstructions. The square-root-transformed, normalized return intensity of the points visually separated stems and leaves in the reconstructed corn canopies (Figure 4). In the corn-thinning experiment, plants were approximately 0.381 m from the LiDAR center. At this lateral distance, given the spread of multi-beam LiDAR’s 16 scanning planes, a fixed plant feature could first enter the LiDAR field of view approximately 0.38 m before the sensor reached it and remain visible until 0.14 m after the sensor passed. Thus, a single plant feature could be sampled over approximately 0.52 m of cart travel.

### 3.2. Multi-Beam Scanning

Downsampled point clouds were used to test whether angled scan planes detected more canopy structure than the zero-degree scan plane alone. For each plot, points from the aggregated angled scan planes were randomly downsampled to match the number of points collected by the corresponding zero-degree scan plane. This allowed voxel occupancy to be compared between scan-angle classes while controlling for differences in point-cloud density.

**Figure 5:**
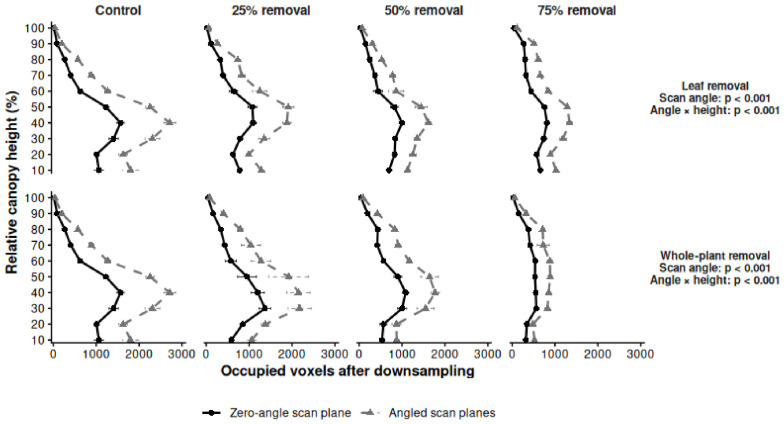
Vertical distribution of occupied voxels from zero-degree and angled LiDAR scan planes in corn leaf-removal and whole-plant-removal treatments. Point clouds from the aggregated angled scan planes were randomly downsampled to match the number of points collected by the corresponding zero-degree scan plane. Voxel counts were then summarized by relative canopy height to compare how scan-angle class affected vertical canopy detection after controlling for point-cloud density. Separate profiles are shown for leaf-removal and whole-plant-removal treatments across removal amounts. Higher voxel counts from the downsampled angled scan planes indicate that multi-angle sampling detected additional canopy volume beyond that captured by the zero-degree scan plane alone.

A three-way ANOVA tested the effects of removal amount, scan-angle, canopy height, and their interactions on occupied voxel count. In the leaf-removal experiment, scan-angle class had a significant effect on voxel count, and the scan-angle class × canopy-height interaction was also significant (Figure 5). In all leaf-removal treatments, the downsampled angled scan planes produced a total of 1.7 times more occupied voxels than the zero-degree scan plane. The scan-angle class × canopy-height interaction indicated that the difference between zero-degree and angled-beam sampling varied with canopy position, with the largest relative differences occurring in the upper canopy.

A similar pattern was observed in the whole-plant-removal experiment. Scan-angle class had a significant effect on voxel count, and the scan-angle class × canopy-height interaction was significant. Across whole-plant-removal treatments, the downsampled angled scan planes produced 1.78 times more occupied voxels than the zero-degree scan plane. As in the leaf-removal experiment, the effect of scan-angle class changed with canopy height, indicating that angled beams altered the vertical distribution of detected canopy volume (Figure 5).

Together, these results support the idea that multi-angle LiDAR sampling provides additional canopy-interception pathways compared with a single perpendicular scan plane. Although point density was controlled by downsampling, the angled scan planes still detected more occupied canopy volume, particularly at higher relative canopy positions where occlusion from lower leaves and stems would be expected to limit a single zero-degree scan plane.

### 3.3. LiDAR-Derived Traits Distinguished Plant Removal from Leaf Removal in Corn

Persistent homology-based stand count was applied to filtered corn point clouds to determine whether the LiDAR data could detect changes in plant number. Before topology analysis, point clouds were filtered to retain points between 0.1 and 1.0 m above the ground. The lower threshold removed ground returns and other near-surface noise, while the upper threshold excluded the more diffuse upper canopy, where leaves were less useful for identifying individual plant positions. Points were also filtered by relative return intensity to emphasize lower-canopy stem and leaf material associated with individual plants.

**Figure 6:**
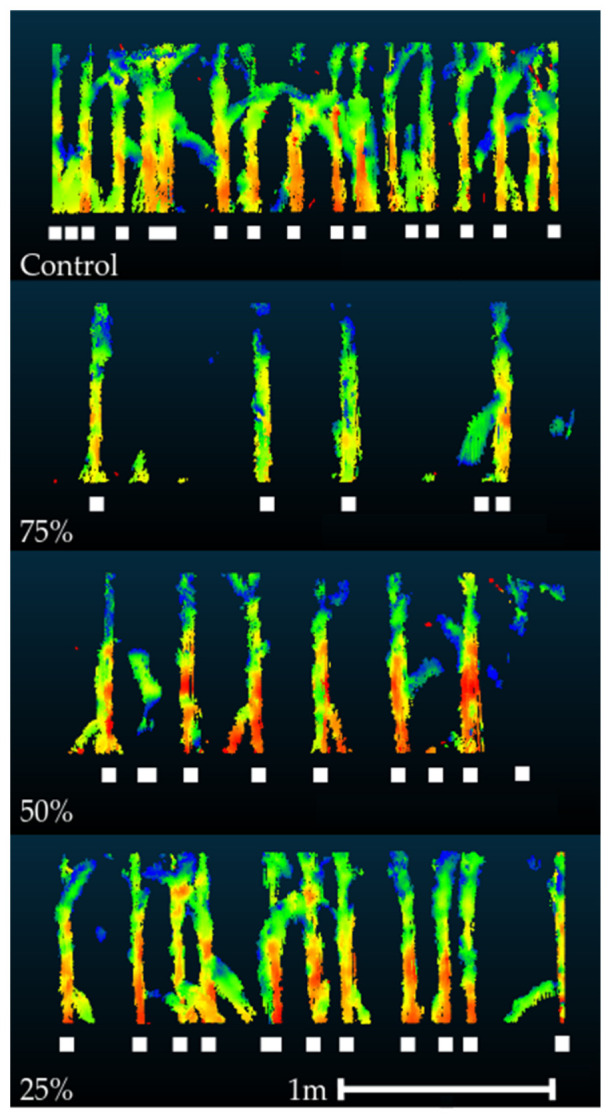
Representative filtered corn point clouds used for persistent homology-based stand count estimation. Point clouds were filtered by height, retaining points between 0.1 and 1.0 m above the ground, and by reflective intensity before analysis with the persistent homology algorithm. This preprocessing emphasized lower-canopy stem structures while reducing interference from leaves and upper-canopy points. White squares indicate plant locations detected by the topology algorithm. The examples show a control plot and plots with 25%, 50%, and 75% plant removal, illustrating the reduction in detected plant number as removal intensity increased. Colors represent the square-root transformation of normalized LiDAR remissivity where blue is lower intensity points and red is higher intensity.

The resulting point clouds preserved the spatial structure of the lower canopy and allowed the topology algorithm to identify plant locations within each plot (Figure 6). In representative plant-removal plots, the number of detected plant locations decreased as removal intensity increased, indicating that the filtered point clouds retained enough stem-associated structure for persistent homology-based stand-count estimation.

**Figure 7:**
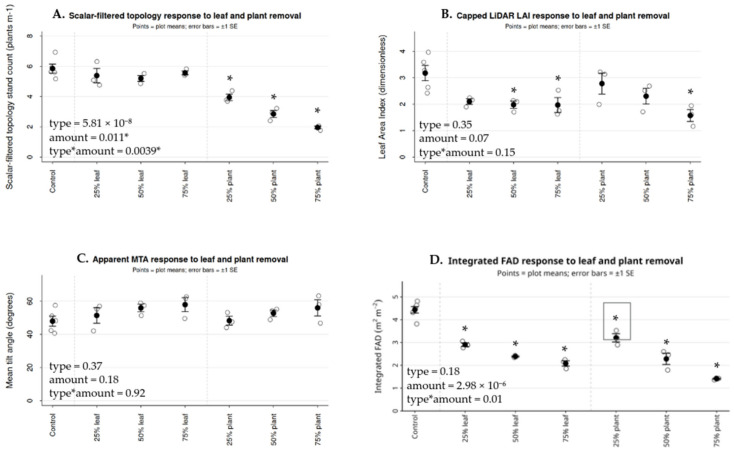
LiDAR-derived architectural traits detected canopy structural responses to leaf and plant removal. Scalar-filtered topology stand count was estimated using a persistent homology algorithm applied to reflectance-filtered point clouds. LiDAR apparent leaf area index (LAI) was estimated from gap fraction across scan angles, analogous to optical hemispherical LAI approaches. Mean tilt angle (MTA) was estimated from the angular dependence of LiDAR gap fraction and represents the apparent average inclination of canopy elements. Integrated foliage area density (FAD) was calculated by estimating apparent FAD within vertical canopy layers and summing FAD_i_ × Δy_i_ across a fixed vertical window, producing an LAI-like area-per-ground-area trait that retains information from the vertical FAD profile. Points represent plot means, and error bars represent ±1 SE. Treatment effects were evaluated by ANOVA for each trait, and Tukey’s honestly significant difference test was used for multiple comparisons. An asterisks * indicates that treatments significantly different from the pooled control at α = 0.05.

The type of material removed, leaf or whole-plant, had a significant effect on topology-based stand count (Figure 7A) There was also a significant removal type × removal amount interaction (Figure 7A). This interaction was driven by differences in the response of stand count to the two types of removal. None of the leaf-removal plots had stand counts that differed significantly from the control, whereas all the plant-removal plots had significantly lower stand counts than the control.

LiDAR based estimations of LAI detected reductions in canopy material, with significant treatment effects associated with the removal amount (Figure 7B). Thus, for both plant-removal and leaf-removal treatments, the amount of material removed had a significant effect on LAI. However, contrast statements showed that LAI did not differ significantly among removal levels within either removal type. For example, LAI values in the 25%, 50%, and 75% plant-removal plots did not differ significantly from one another.

Mean tilt angle was not significantly affected by the thinning treatments (Figure 7C). In contrast, both removal type and removal amount significantly affected plot-bound FAD (Figure 7D). The removal type × removal amount interaction was also significant, indicating that the effect of removal amount depended on which type of material was removed. This interaction was driven by a larger reduction in integrated FAD in the plant-removal treatments than in the leaf-removal treatments.

The multiple LiDAR traits captured by a single scan were able to identify two key aspects of canopy structure: the number of plants and the amount of leaf material. Persistent homology-based stand count was reduced by plant removal but not by leaf removal. The plot-bound FAD measurements were able to detect differences in leaf area in the leaf-removal plots.

To explore method variability, the coefficient of variation was calculated for each plot from the five repeated scans (Table 1). Plot 1 was excluded from this analysis because steep local slope produced poor scan geometry. Across all treatments, LiDAR-derived LAI had an overall CV of 5.5 ± 0.5%, with plot-level CVs ranging from 1.7% to 10.5%. Across all treatments, LiDAR-derived MTA had a CV of 2.6 ± 0.3%, with plot-level CVs ranging from 0.3% to 5.3%. Across all treatments, integrated FAD had an overall plot-level repeatability CV of 4.7 ± 0.6%, with plot-level CVs ranging from 1.3% to 11.1%. Topology-derived stand count had an overall plot-level repeatability CV of 12.2 ± 1.1%, with plot-level CVs ranging from 4.5% to 23.5%.

## 4. Discussion

This study demonstrates that a single-operator, push-cart LiDAR platform can collect synchronized multi-beam LiDAR, encoder, and IMU data and that data can be processed into biologically meaningful plot-level canopy traits. In hairy vetch, voxelized canopy volume was strongly associated with destructively measured dry biomass, showing that the cart and processing workflow captured differences in whole-plant size across a range of biomass values. In corn, point clouds supported additional structural traits, including persistent homology-based stand count, LAI, mean tilt angle, and plot-bounded integrated FAD. This flexibility is one of the platform’s main strengths: a single scan produces a reusable 3D archive of the plot, and different algorithms can then extract traits relevant to biomass, stand density, canopy architecture, or vertical leaf distribution.

The multi-beam LiDAR produced better data for use in row crops. In the corn thinning experiment, after the angled beams were downsampled to the same point density as the zero-degree scan plane, the vertical distribution of occupied voxels was greater in the angled-beam scan. Thus, the multi-beam lidars also offer a greater overall volume of scans, along with a variety of angles that increase transmittance through the canopy. This is important because many new multi-beam LiDAR scanners are available and this makes it clear that phenotyping systems should favor multi-beam models when available. In corn, lower leaves and stems can intercept the perpendicular beam before it reaches higher canopy layers. Multi-angle sampling reduced this limitation by allowing the sensor to observe canopy material from multiple directions during a single pass. However, multi-beam scanning does not eliminate occlusion in dense row crops. The upper canopy remained less densely sampled than the lower canopy. This can be seen in the control plot of the corn example point cloud (Figure 4). There are points that reach the upper canopy but no instances where you can see a continuous feature that could be identified as a leaf.

The LiDAR-derived traits captured distinct elements of the canopy structure. Persistent homology-based stand count accurately recovered the plant number. The known field planting density was approximately 6.5 plants m^−1^, and the topology estimate was consistently near 6 plants m^−1^ in the control and leaf-removal plots. Stand count responded strongly to plant removal but was insensitive to leaf removal, indicating that the lower-canopy, intensity-filtered topology workflow detected stem-associated structures rather than total canopy material. LAI tracked the overall reduction in canopy material but did not distinguish whether the removed material was leaves or whole plants, declining with removal amount regardless of removal type.

The stand count algorithm was calibrated by tuning parameters to produce a plant density in the control plots that matched the known field planting density. The persistence threshold, point-cloud height range, and intensity-filtering settings were selected after a series of calibration attempts. Some degree of supervision may always be needed for persistent homology-based plant counts, especially when absolute accuracy is required rather than detecting relative differences among treatments. One practical strategy is to define a default range of candidate settings for persistence, height range, and intensity filtering, generate multiple stand count estimates, and select the combination that produces a value closest to the known field density. The algorithm is most likely to fail at high planting densities, where neighboring plants blur together and become difficult to distinguish as separate features. Therefore, if the method can be validated in the highest-density plots, there should be greater confidence that it will identify plots with lower plant densities.

If the thinning treatments had any effect on mean tilt angle, it was expected that they would increase the mean tilt angle. The LiDAR scans would have more points associated with the upper portion of the corn canopy where the leaves and features are more erect. However, LiDAR estimations of mean tilt angle were not sensitive to the imposed treatments. MTA was estimated using the same general approach as a hemispherical analyzer, where leaf inclination is inferred from the angular dependence of gap fraction across five fixed zenith rings. With only five angular bins used to calculate MTA, the leaf-angle inversion may be weakly constrained. Unlike the optical sensors, the LiDAR data are not limited to five optical rings because the zenith angle of each beam is known for every return. Future work could use this finer angular sampling to develop a LiDAR-specific leaf-angle model, potentially estimating a fuller or more accurate leaf-angle distribution.

A key advantage of the FAD approach is that it directly uses distance information from the LiDAR scans to define and analyze only the portion of the scan considered part of the plot. For many scientific field plots, the 30-m gap distance or outer range of a scanning instrument is much larger than the experimental plot being measured. This creates a practical problem for LAI-style gap-fraction methods. If the sensor can see outside the plot, then open alleys, missing neighboring plants, or the edge of the planted field, that becomes part of the LAI estimate. In the corn experiment, the lowest LAI and MTA values were typically observed in plots located on the outer edge of the experiment, suggesting that the LiDAR was seeing beyond the experimental canopy and into open areas outside the planted field. This likely lowered those estimates artificially and illustrates a consequence of applying a whole-view gap-fraction algorithm to small experimental plots.

FAD directly addressed this issue by constraining the analysis to the plot-bounded volume. Instead of asking only whether a ray was intercepted, the FAD algorithm used both where each LiDAR ray traveled and how far it traveled before interception. Each ray was evaluated only within the defined experimental plot, so returns and path lengths outside the plot did not contribute to the estimate. FAD also addressed limitations associated with applying gap-fraction LAI methods to LiDAR data. The LAI algorithm could only evaluate the gap fraction across the 90° field of view on either side of the sensor and could not measure canopy material below the sensor height. Because the LiDAR was mounted approximately 0.40 m above the ground, LAI could miss changes in lower-canopy material below that height. In contrast, FAD was estimated within vertical layers and allowed a user-defined minimum height. In this study, the minimum FAD height was set to 0.1 m to exclude ground returns while still capturing lower-canopy material. Together, the plot-bounded geometry and vertical layering made FAD more sensitive to the leaf- and plant-removal treatments than the whole-view LAI and MTA approaches.

For treatment comparisons, FAD was summarized as an integrated value by summing layer-specific apparent FAD multiplied by layer thickness. This produced an LAI-like area-per-ground-area trait while retaining the option to examine the underlying vertical FAD profile. These profiles could identify where canopy material was removed or redistributed. For example, leaf removal would be expected to change the vertical profile of the leaf area differently than whole-plant removal. Formal height-by-treatment contrasts were not explored here because the experiment had limited replication and the profile analysis would require more complex models than were appropriate for this methodological study. However, vertical FAD profiles are a major advantage of the LiDAR approach and should be explored in future studies. It could be used to detect treatment effects like senescence in the lower canopy due to drought.

Cart speed is another operational variable that should be evaluated in future validation studies. At a fixed LiDAR scan frequency, faster cart movement increases the distance traveled between successive LiDAR rotations and reduces the number of returns collected per unit length of row. As a result, excessive travel speed could reduce point density, increase gaps in the point cloud, and increase variability in derived traits, especially traits that depend on fine-scale spatial structure such as topology-derived stand count. In this study, cart speed was not experimentally varied, but the low scan-to-scan coefficients of variation for LAI, MTA, and integrated FAD suggest that the cart was operated within a reasonable speed range for these continuous canopy traits.

The lack of treatment response in MTA should not be interpreted as evidence that LiDAR cannot estimate canopy inclination. Instead, it shows that this MTA implementation was not sufficient for this crop and experiment, especially considering the fact that it only had three statistical replicates. One way that MTA could be improved would be to make the analysis constrained to the plot area, like FAD. The MTA algorithm used here was similar to traditional gap fraction LAI in that it was not constrained by plot size. Thus, the potential effects of the treatment would be influenced by plots outside the four experimental rows that did not have plants or leaves removed. Of the three statistical replicates measured, the “low” value for the MTA plots in each experimental treatment can be tracked back to the first row of plots measured. The first row was closest to the edge of the field. The beams that would have exited the plots exited into the field and artificially lowered the MTA. The results from the multi-beam analysis show that as leaves and plants were removed, the lidar saw a greater proportion of material in the upper portion of the canopy. The material in the upper portions of the canopy includes the stems and the more vertical leaves. Therefore, regardless of the type of material removed, leaves or whole plants, MTA should increase as more plants or leaves are removed. Perhaps without the edge effects of first rows, MTA would follow this trend where there was a significant effect of removal amount but not type. Future MTA algorithms may need to use a wider angular range, more flexible fitting approaches, and the full distance-aware ray information rather than relying on angular bins alone.

Although multi-beam sensors offer better penetration into the upper canopy, there were still practical limitations. The angled scan planes observe the same plant feature over an extended distance of cart travel, so wind or plant movement can blur or duplicate structures in the reconstructed point cloud. This limitation can be pronounced in tall, flexible canopies like corn. Beam-specific differences in angular resolution and return intensity may also affect point density and filtering. Some scanning-planes produce denser or higher-intensity returns than others, and filtering steps can therefore remove points from some beams more aggressively than others. Future versions of the workflow should include more aggressive IMU correction, improved beam weighting for intensity normalization, or scan-plane calibration.

In conclusion, the results show that multi-beam LiDAR can provide a practical and improved approach for field phenotyping. The cart produced plot-level point clouds in two very different crop systems, and the data from a single plot in the corn-thinning experiment supported biomass estimation, vertical canopy profiling, stand-count estimation, LAI, MTA, and integrated FAD. FAD is a particularly interesting advance because it improves the ability of scientists to make inferences about small or irregular shaped plots. LiDAR was able to make the important distinction between a plot that had lost leaves or plants, which is important for interpreting results. For instance, the proximal cart is particularly suited to detecting lower canopy elements and can measure leaf area at varying height intervals. It would be well suited to detect the loss of lower leaves in a plant canopy affected by drought. As algorithms improve, the value of those archived point clouds will increase. Future work should focus on validating topology-derived stand counts against manual plant counts, validating FAD against destructive or independently measured leaf-area profiles, automating parameter selection, and testing the workflow across crop species, planting densities, and growth stages.

## Figures and Tables

**Table 1:** Scan-to-scan repeatability of LiDAR-derived canopy traits. Values are treatment-level summaries of plot-level coefficients of variation calculated across repeated scans and are presented as mean CV ± SE, with the plot-level CV range in parentheses.

Treatment	n Plots	LAI CV (%)	MTA CV (%)	Integrated FAD CV (%)	Stand Count CV (%)
Control	5	4.8 ± 1.3 (2.1–8.5)	2.1 ± 0.5 (1.1–4.2)	4.1 ± 1.2 (1.3–7.5)	15.1 ± 2.5 (7.8–21.5)
25% leaf removal	3	6.9 ± 0.5 (5.9–7.6)	3.1 ± 0.8 (2.0–4.7)	7.0 ± 1.6 (5.2–10.1)	13.6 ± 2.9 (8.7–18.6)
50% leaf removal	3	7.8 ± 2.2 (3.5–10.5)	2.9 ± 0.6 (2.0–4.2)	6.7 ± 2.3 (3.3–11.1)	9.8 ± 0.9 (8.6–11.4)
75% leaf removal	3	3.9 ± 1.2 (1.7–5.5)	1.9 ± 1.1 (0.3–3.9)	3.8 ± 0.7 (2.5–4.9)	6.4 ± 1.2 (4.5–8.7)
25% plant removal	3	7.1 ± 1.2 (5.6–9.6)	3.1 ± 0.6 (2.0–4.1)	4.5 ± 2.5 (1.5–9.6)	16.9 ± 4.0 (9.7–23.5)
50% plant removal	3	3.9 ± 1.1 (2.5–6.0)	2.7 ± 0.9 (1.2–4.3)	2.6 ± 0.5 (1.8–3.4)	13.0 ± 3.7 (5.8–17.8)
75% plant removal	3	4.5 ± 1.2 (2.8–6.7)	2.8 ± 1.4 (0.4–5.3)	4.6 ± 1.0 (2.7–6.4)	8.5 ± 1.2 (7.1–10.8)

## Data Availability

The data that support the findings of this study are available from the corresponding author upon reasonable request. The data are not publicly available due to institutional considerations but can be provided to qualified researchers for the purpose of replicating or extending the analysis.
